# Do We Need More Structured MD Thesis Programs? A Propensity Score Matched Analysis of the Research Program at the Medical Faculty Dresden

**DOI:** 10.1007/s40670-024-02077-x

**Published:** 2024-06-13

**Authors:** Jean-Paul Bereuter, Mark Enrik Geissler, Anna Klimova, Rona Geissler, Corina Oswald, Ali El-Armouche, Katja El-Armouche, Lydia Günther, Andreas Deußen

**Affiliations:** 1 Department of Visceral, Thoracic and Vascular Surgery, Medical Faculty and University Hospital Carl Gustav Carus, Technische Universität Dresden, Fetscherstraße 74, 01307, Dresden, Germany; 2 Institute for Medical Informatics and Biometry, Medical Faculty and University Hospital Carl Gustav Carus, Technische Universität Dresden, Dresden, Germany; 3 Department of Physiology, Medical Faculty Carl Gustav Carus, Technische Universität Dresden, Dresden, Germany; 4 Institute of Pharmacology and Toxicology, Medical Faculty and University Hospital Carl Gustav Carus, Technische Universität Dresden, Dresden, Germany; 5 Division of Medical Biology, Department of Psychiatry and Psychotherapy, Medical Faculty and University Hospital Carl Gustav Carus, Technische Universität Dresden, Dresden, Germany

**Keywords:** Medical research education, Structured doctorate program

## Abstract

**Introduction:**

Conducting a Medical Doctorate (MD) thesis is desired by the majority of medical students. However, the needed scientific competencies are not regularly implemented in medical education. To support students during their MD thesis, a graduate college was implemented. The present study aims to investigate the impact of this structured MD thesis program on the outcome of the MD thesis and the further scientific career.

**Methods:**

An online survey covering 59 items was distributed to all current and former medical students who officially started their MD thesis from 2011 to 2022. The survey investigated the impact of the structured MD thesis program on the scientific development of participating students compared to students outside the structured program.

**Results:**

Based on a total of 370 complete answers, the analysis indicated that participants of the structured program have a significantly better outcome of their MD thesis compared to the control cohort based on objective parameters such as the thesis grade, the number of first-author publications, attendance of congresses, and the number of rewards. Additionally, participation in the program led to a more sustainable integration of students into research measured by the participation or pursuit of clinician scientist programs. Propensity score matched analyses of 60 participants confirmed the results.

**Conclusion:**

Participation in a structured MD thesis program significantly improved the outcome and may support sustainable integration into research. Therefore, the implementation of such programs should be further expanded to secure the education of scientifically trained MD graduates.

**Supplementary Information:**

The online version contains supplementary material available at 10.1007/s40670-024-02077-x.

## Introduction

The medical field is constrained by a plethora of challenges such as the implementation of new technologies, the exponential increase of medical knowledge, pandemics, personalized medicine, and an ageing population [1–5]. To drive change and overcome these upcoming and existing challenges, education of medical students is of utmost importance. Medical education at universities forms future generations of physicians who will be confronted by these challenges [6, 7].

Besides clinical duties, research is of great importance to drive medicine forward. However, in the current medical curriculum in Germany, research is marginalized [8, 9]. The most common way to contribute to research and acquire basic research skills is still the conduct of a doctoral thesis [9, 10]. Of note, the term Dr. med. (MD) may only be carried after the conduct of a medical dissertation in addition to completion of medical school whereas in the United States (US) it is awarded upon completion of medical school. The requirements which need to be fulfilled for acceptance of a medical dissertation are designed by each medical school individually and vary throughout Germany [11, 12]. Nevertheless, most students desire an MD title. However, compared to other scientific subjects and on an international level, the MD is often regarded to be of less scientific complexity and lower content, yet among medical professionals it still bears a high connotation of medical and scientific knowledge [10, 13, 14]. While this may reflect different approaches between academic faculties, it may also prompt raising questions about the effectiveness of the current system in educating future physicians capable of making significant contributions to medical research, advancing healthcare and ensuring patient safety [8, 15].

Facing the lack of a consistent nationwide curriculum concerning scientific education, medical students frequently express a desire to acquire more scientific competencies during their medical studies [8]. Certain medical schools already implemented scientific curricula and research projects [16–18]. One relatively new example for implementing scientific education at the Medical Faculty of Dresden (MFD) is the Carus Promotionskolleg Dresden (CPKD), a structured MD thesis program for experimental research projects [19]. Other universities such as the Medical Faculty of the University of Cologne developed and established a systematic science curriculum in previous years. The implementation of this research program was well accepted and resulted in a higher number of conducted research projects as well as higher numbers of accepted grant applications [16]. The research programs implemented in Germany can be seen equivalent to MD/PhD programs in the US [11, 20]. Although there are differences between the MD and the PhD concerning scientific complexity and content, both support their participants in gaining scientific knowledge and experience [21, 22]. Therefore, both programs help to improve high scientific quality and recruitment of future generations of clinician scientists [21, 23].

The concept of CPKD that was followed at the MFD was the implementation of a structured MD thesis program for experimental research projects. Within this program, students are supported both scientifically and financially for 1 year during their MD thesis. The scientific support contains theoretical lectures for good scientific practice and theories of biomedical science as well as practical components such as project planning, abstract/poster design, and data presentation [19]. Students participating in this CPKD program pause their regular medical curriculum for a period of usually 1 year. For this period, they obtain separate funding via the CPKD because federal funding excludes support of medical students for activities outside the regular curriculum.

Although major scientific foundations such as the Else Kröner Fresenius Foundation supported these initiatives for more than 10 years, scientific evidence that proves an objective benefit of such programs is limited to date [24]. In part, this relates to the time course, which is required to gain robust surrogate data on the success of a particular program. This study investigates the impact of a structured MD thesis program, based on an 11-year experience on the example of CPKD on the outcome of the MD thesis. It also investigates the interest of CPKD participants compared to a control cohort for subsequent participation in a clinician scientist program.

The CPKD is a structured program supporting students during the conduct of their medical thesis [19]. The program was introduced in 2011 as the Else Kröner Fresenius Promotionskolleg Dresden (EKPK). After the full funding period by the Else Kröner Fresenius Foundation ended after 6 years in 2017, the program was continued by the medical faculty and renamed CPKD. To select projects and participants, the program has a stepwise selection process. Researchers of the MFD and partner institutions submit research proposals for a doctoral thesis project. In a first step, a committee consisting of three board members of the CPKD evaluates the submitted projects. After approval of a proposed project, students are allowed to apply for these positively selected research projects. The program selects approximately 15 students annually. Ten projects are funded per year by the faculty and typically around five further projects receive support by external funding. Students are given the task to present their anticipated project and a publication in the respected field, which is attributed to them 1–2 weeks in advance. The presentations are assessed by at least three board members of the CPKD. Results from this assessment are ranked and final individual selection for participation is based on the rank order of applicants. This final decision is obtained by agreement of the entire CPKD board. After selection into the program, students embark on a full-time 12-month scientific journey while pausing their medical studies. During this year, students are requested to participate in the mandatory education program covering basics of scientific work and research conduct. Besides theoretical aspects, e.g., theories and history of science as well as good scientific practice, practical contents are taught, for instance abstract writing, poster composition, and presentation skills. Simultaneously, students perform research work on their projects. The CPKD emphasizes scientific exchange and group activities throughout the program, empowering students to exchange ideas and form long-lasting connections. During the 12-month program, an advisory committee, consisting of at least three senior scientists knowledgeable in the specific project, is requested to meet at least three times with the student. Students are also advised by mid-career scientists during their daily work. At the end of the program, there is an oral presentation as well as poster competition and a poster prize given to the best presenter. After completing the program, students gain alumni status and support younger fellows as mentors. The CPKD established strong connections to existing clinician scientist programs to support the future career of their members.

## Materials and Methods

### Survey Design

This cross-sectional study was performed from January to March 2023 using the online survey tool Lime Survey (https://www.limesurvey.org/de/). The survey design was conducted by the authors in an iterative collaborative process. All survey items were reviewed by experts in the field from our faculty. All questions were further evaluated by medical students to assess the required time as well as to identify and remove ambiguities from the questionnaire. The answers collected during this review process were not included in the final data analysis.

The questionnaire consisted of 59 different items. We requested biographic participant information (12 items) and data regarding the medical studies (7 items). In addition, students were asked for information concerning their MD thesis (14 items). Students who participated in a structured MD thesis program were requested for data regarding their attendance of the program (10 items). Due to the relevance of the COVID-19 pandemic at the time of survey, students were also asked for the subjective impact of the pandemic on their MD thesis project (3 items). Moreover, information concerning the students’ current/future research (5 items) as well as their scientific output (8 items) were requested. The participants needed approximately 15 min to answer all questions of the survey.

### Distribution and Study Cohorts

The survey was distributed via email to medical students and physicians either performing (*N* = 1949) or having performed (*N* = 1471) their medical studies and MD thesis at MFD and the University Hospital Carl Gustav Carus Dresden from 2011 to 2022. Students and alumni of the MFD were assigned to either the control group or the intervention group. The intervention group included students who participated in the CPKD, either full or part time. Due to the fact that students could conduct research projects that were associated with the CPKD program, part-time participation in the program is possible. The control group, comprising students not enrolled in the CPKD program, conducts their MD thesis projects without the structured support provided by the CPKD program. This includes the absence of financial aid and dedicated scientific guidance from a program-associated scientific committee. Nevertheless, these students have access to scientific supervisors and pursue their research projects within the standard academic framework available to all medical students.

### Participants and Data Protection

Only medical students and physicians who officially started conducting their thesis by registering their thesis at MFD were allowed to participate in the study. All study participants agreed to participation and to data protection. Questionnaire responses were saved and stored on servers of TU Dresden. This study follows the data privacy rules of the MFD as well as Technical University of Dresden. Considering the non-interventional, anonymized nature of the survey, our study was not subject to the conventional requirements for review and approval by an Institutional Review Committee for Human Use.

### Data Treatment and Analysis

Statistical analysis was conducted using SPSS version 28 (IMB Corp, Armonk NY, USA). Continuous variables were summarized as mean values and standard deviations (SDs) or median and interquartile range. Discrete variables were summarized as absolute and relative frequencies. Depending on the data characteristics, the appropriate statistical test was utilized (independent samples Student’s *t*-test, chi-square test) to conduct between-group comparisons. In addition, the propensity score matching procedure was applied to match the study groups, once by age and once by the type of an experimental thesis. The between-group differences were assessed using the matched samples. A *p*-value less than 0.05 was considered statistically significant.

## Results

### Sample Characteristics

Out of 3420 preliminarily contacted potential participants, 440 could not be reached via email due to invalid email addresses, leaving a potential pool of 2980 subjects. A total of 370 responded and were included for further analyses (CPKD: *N* = 60; ctrl: *N* = 310; response rate, 12.4%). The average participant age was significantly lower in the CPKD compared to the control group (CPKD: 27 years vs. ctrl: 32 years; *p* < 0.001). Regarding the distribution between male and female, no significant differences were observed between both groups (CPKD: 46.7% female vs. ctrl: 63.8% female; *p* = 0.104). The majority of participants performed an experimental thesis, followed by clinical and theoretical thesis projects. Experimental thesis projects were significantly more often performed by CPKD students compared to the control group (CPKD: 96.7% vs. ctrl: 27.1%; *p* < 0.001). In contrast, clinical thesis projects were significantly less often performed by students in the CPKD compared to the control group (CPKD: 5% vs. ctrl: 57.1%; *p* < 0.001). There were no significant differences observed with respect to the professional career of parents working in medical fields or the educational background between both groups. Nevertheless, students in the CPKD group had significantly higher high school grade point average (HS-GPA; ranging from 1.0 = very good to 4.0 = sufficient) compared to the control group (CPKD: 1.33 vs. ctrl: 1.61; *p* < 0.001). Additionally, the GPA for the first state examination (SE-GPA; ranging from 1.0 = very good to 4.0 = sufficient) was significantly higher in the CPKD group compared to the control (CPKD: 2.19 vs. ctrl: 2.54; *p* < 0.001) (Table 1).

**Table 1:** Basic participant characteristics

	CPKD (*N* = 60)	Control (*N* = 310)	*p*-value
Age, mean [years (IQT)]	27 (25, 29)	32 (27, 36)	**< 0.001**
Sex [*N* (%)]			0.104
Male	32 (53.3)	109 (34.9)	
Female	28 (46.7)	199 (63.8)	
Diverse	0 (0)	2 (0.6)	
Thesis [*N* (%)]			
Theoretical	2 (3.3)	49 (15.8)	**0.018**
Clinical	3 (5)	177 (57.1)	**< 0.001**
Experimental	55 (96.7)	84 (27.1)	**< 0.001**
Biomedical background of parents [*N* (%)]	28 (46.7)	118 (38.1)	0.27
Education background of parents [*N* (%)]	16 (27.1)	107 (35.3)	0.287
HS-GPA [mean (SD)]	1.33 (0.4)	1.61 (0.52)	**< 0.001**
SE-GPA [mean (SD)]	2.19 (0.56)	2.54 (0.83)	**< 0.001**
Number of supervisors (> 2) [*N* (%)]	18 (30)	39 (12.6)	**< 0.001**

Significant *p*-values are highlighted in bold

*IQT* interquartile range, *HS-GPA* high school grade point average ranging from 1.0 = very good to 4.0 = sufficient, *SE-GPA* state examination grade point average ranging from 1.0 = very good to 4.0 = sufficient

### Impact on Students’ Satisfaction and Scientific Output

Throughout the conduct of their medical thesis, CPKD students had on average more supervisors compared to the control group (> 2 supervisors; CPKD: 30% vs. ctrl: 12.6%; *p* < 0.001). Regarding students’ satisfaction with their supervisors, daily work during the thesis, and outcomes, there were no significant differences observed between both groups. The majority of CPKD students would conduct their thesis again (76.7%). However, participants of the program did not rate (1–10; 1 = not relevant; 10 = very relevant) the relevance of the MD thesis higher compared to the control group (CPKD: 5.93 vs. ctrl: 4.89; *p* = 0.244) (Table 2).

**Table 2:** Impact of the CPKD on the subjective outcome of the doctoral thesis

	CPKD (*n* = 60)	Control (*n* = 310)	*p*-value
Satisfaction supervision [mean (SD)]	6.93 (3.14)	6.49 (3.36)	0.345
Satisfaction daily work [mean (SD)]	6.92 (2.77)	6.29 (2.87)	0.12
Satisfaction outcome [mean (SD)]	6.70 (3.03)	6.73 (2.94)	0.944
Conduct thesis again [mean (SD)]	46 (76.7)	196 (63.2)	0.064
Relevance MD thesis [mean (SD)]	5.93 (2.96)	4.89 (2.87)	0.244

CPKD students published on average more papers as first authors compared to the control group (CPKD: 0.62 vs. ctrl: 0.33; *p* = 0.017), while the average number of co-author publications was not significantly different between both groups (CPKD: 0.8 vs. ctrl: 0.48; *p* = 0.084). In addition, participants of the CPKD attended congresses significantly more often in comparison to the control group (CPKD: 68.3% vs. ctrl: 21.6%; *p* < 0.001). They also achieved more recognition and gained significantly more awards for their scientific work compared to the control group (CPKD: 38.3% vs. ctrl: 4.2%; *p* < 0.001) (Table 3).

**Table 3:** Impact of the CPKD on the objective outcome of the doctoral thesis

	CPKD (*N* = 60)	Control (*N* = 310)	*p*-value
First-author publications [mean (SD)]	0.62 (1.51)	0.33 (0.67)	**0.017**
Co-author publications [mean (SD)]	0.80 (1.77)	0.48 (1.20)	0.084
Attendance congresses [*N* (%)]	41 (68.3)	67 (21.6)	**< 0.001**
Rewards [*N* (%)]	23 (38.3)	13 (4.2)	**< 0.001**
Thesis grade [mean (SD)]	1.6 (0.53)	2.3 (0.64)	**< 0.0001**

Significant *p*-values are highlighted in bold

The scientific output of CPKD students was further reflected when analyzing the results of their thesis. Therefore, we analyzed data, which had been archived by the faculty with respect to the individual thesis grades awarded to medical dissertation students between 2011 and 2022. The anonymized dataset revealed that the average thesis grade awarded differed significantly between participants of CPKD and control group (CPKD: 1.6 vs. ctrl: 2.3; *p* < 0.0001, *n*1 = 64, *n*2 = 1679) (Table 3). Further, we analyzed the dataset with respect to individual thesis grades. The analysis of all three thesis types (experimental, clinical, and operative) showed that CPKD students significantly more often received the grade *summa cum laude* compared to the control group (CPKD: 41.8% vs. ctrl: 7.7%; *p* = 0.0094). While the grade *magna cum laude* was not differing between both groups (CPKD: 57.4% vs. ctrl: 55.7%; *p* = 0.85), CPKD students received significantly less often the grade cum laude (CPKD: 0.8% vs. ctrl: 35.7% ; *p* = 0.0048 ) and *rite* (CPKD: 0% vs. ctrl: 1.3%; *p* = 0.0161) (Supplementary Fig. 1).

### Impact on Current Scientific Work

The last section of the questionnaire focused on the current conduct of scientific work. Half of the CPKD students stated that they are still conducting scientific projects. This is significantly more compared to students of the control group who are less engaged in ongoing research projects after finishing their thesis (CPKD: 50% vs. ctrl: 23.2%; *p* < 0.001). In line with this, former participants of the CPKD group continue their scientific education significantly more often in Clinician Scientist Programs (CSPs) in contrast to participants of the control group (CPKD: 11.7% vs. ctrl: 2.6%; *p* = 0.004). In addition, significantly more students of the CPKD group pursue a career as a clinician scientist (CS) compared to the control group (CPKD: 45% vs. ctrl: 10%; *p* < 0.001). Interestingly, the number of publications, including first and co-author publications, after the conduct of their thesis, did not vary significantly between both groups (CPKD: 85% vs. ctrl: 83.2%; *p* = 0.882) (Table 4).

**Table 4:** Impact of the CPKD on the sustainable integration of MD students into the field of experimental research

	CPKD (*N* = 60)	Control (*N* = 310)	*p*-value
Current projects [*N* (%)]	30 (50.0)	72 (23.2)	**< 0.001**
Participation CSP [*N* (%)]	7 (11.7)	8 (2.6)	**0.004**
Pursue CSP [*N* (%)]	27 (45.0)	31 (10.0)	**< 0.001**
Publications after thesis [*N* (%)]	51 (85.0)	258 (83.2)	0.882

Significant *p*-values are highlighted in bold

### Propensity Score Matched Analyses

The exploratory analysis of the CPKD and control groups identified a substantial imbalance in their distributions with respect to the conduct of experimental thesis and age, thus making these two variables potential confounders. In order to account for a confounding bias, we applied a propensity score method to match 60 participants of CPKD with two samples of 60 participants each from the control group [25–27]. The first sample was obtained by using the experimental thesis as a matching variable, and the second one was selected based on age. The comparative statistical analyses were performed for each pair of samples separately and are summarized below.

The first matching was performed by the conduct of an experimental thesis. When comparing the propensity score matched groups, the mean age was significantly different between both groups (CPKD: 27 years vs. ctrl: 31 years; *p* = 0.001). While no differences were observed concerning the students’ parental medical background and the educational background, the HS-GPA was differing significantly between the CPKD and the control group (CPKD: 1.33 vs. ctrl: 1.5; *p* = 0.035). In contrast to the non-matched analysis, the SE-GPA was not different between both groups (CPKD: 2.19 vs. ctrl: 2.42; *p* = 0.101). Regarding the number of supervisors, participants of the CPKD program had significantly more often two supervisors and more (CPKD: 30% vs. ctrl: 13.3%; *p* = 0.006). There were no significant differences in terms of students’ satisfaction with the thesis project, their supervision, daily work, or outcomes of the project. Nevertheless, students would conduct their MD thesis again (CPKD: 76.7% vs. ctrl: 61.7%; *p* = 0.114). Concerning the average number of publications, no differences were observed between both groups concerning first-author publications (CPKD: 0.62 vs. ctrl: 0.43; *p* = 0.389) and co-author publications (CPKD: 0.8 vs. ctrl: 0.48; *p* = 0.203). Congresses were significantly more often attended by CPKD students compared to students of the control group (CPKD: 68.3% vs. ctrl: 33.3%; *p* < 0.001). Moreover, students of the CPKD group gained on average significantly more rewards compared to students not participating in the program (CPKD: 38.3% vs. ctrl: 10%; *p* = 0.001). When analyzing the students’ current scientific work, participants of the CPKD program more often state that they currently work on scientific projects compared to students from the control group (CPKD: 50% vs. ctrl: 18.3%; *p* = 0.001). Concerning their future scientific career as a clinician scientist, significantly more CPKD students pursued to participate in CSPs (CPKD: 45% vs. ctrl: 20%; *p* = 0.006). However, the number of students/physicians who already participate in a CSP is not differing between both groups. Similarly, also the average number of publications after the conduct of the MD thesis is not different between the CPKD and the control group (Table 5).

**Table 5:** Propensity score matched analysis based on an experimental thesis

	CPKD (*N* = 60)	Control (*N* = 60)	*p*-value
Age, mean [years (IQT)]	27 (25, 29)	31 (28, 35)	**< 0.001**
Sex [*N* (%)]			
Male	32 (53.3)	30 (50.0)	0.855
Female	28 (46.7)	30 (50.0)	
Diverse	0 (0)	0 (0)	
Thesis [*N* (%)]			
Theoretical	2 (3.3)	2 (3.3)	1.000
Clinical	3 (5.0)	4 (6.7)	
Experimental	58 (96.7)	58 (96.7)	
Biomedical background of parents [*N* (%)]	28 (46.7)	19 (31.7)	0.135
Education background of parents [*N *(%)]	16 (27.1)	14 (24.1)	0.875
HS-GPA [mean (SD)]	1.33 (0.40)	1.50 (0.49)	**0.035**
SE-GPA [mean (SD)]	2.19 (0.56)	2.42 (0.94)	0.101
Number of supervisors (> 2) [*N* (%)]	18 (30.0)	8 (13.3)	**0.006**
Satisfaction supervision [mean (SD)]	6.93 (3.14)	7.20 (2.87)	0.628
Satisfaction daily work [mean (SD)]	6.92 (2.77)	7.15 (2.50)	0.629
Satisfaction outcome [mean (SD)]	6.70 (3.03)	7.12 (2.84)	0.439
Conduct thesis again [mean (SD)]	46 (76.7)	37 (61.7)	0.114
Relevance MD thesis (> 5) [*N* (%)]	36 (60)	24 (40)	0.08
First-author publications [mean (SD)]	0.62 (1.51)	0.43 (0.65)	0.389
Co-author publications [mean (SD)]	0.80 (1.77)	0.48 (0.72)	0.203
Attendance congresses [*N* (%)]	41 (68.3)	20 (33.3)	**< 0.001**
Rewards [*N* (%)]	23 (38.3)	6 (10.0)	**0.001**
Current projects [*N* (%)]	30 (50.0)	11 (18.3)	**0.001**
Participation CSP [*N* (%)]	7 (11.7)	2 (3.3)	0.166
Pursue CSP [*N* (%)]	27 (45.0)	12 (20.0)	**0.006**
Publications after thesis [mean (SD)]	51 (85.0)	49 (81.7)	0.806

The analyses of participant characteristics, subjective and objective outcome parameters, and the sustainable integration in scientific research are depicted. Significant *p*-values are highlighted in bold

To exclude bias that was introduced by the significant difference of age between the CPKD and the control group being found not only in the general analysis (CPKD: 27 years vs. ctrl: 32 years; *p* < 0.001) but also in the first propensity score matched analysis (CPKD: 27 years vs. ctrl: 31 years; *p* = 0.001), another matching based on age was conducted. While no differences were observed concerning the students’ parental medical background and the educational background, the SE-GPA was differing significantly between the CPKD and the control group (CPKD: 2.19 vs. ctrl: 2.5; *p* = 0.02). In contrast to the non-matched and first matched analyses, the HS-GPA was not different between both groups (CPKD: 1.33 vs. ctrl: 1.41; *p* = 0.258). In line with first matching, the number of supervisors as indicated by more than two supervisors per project was significantly higher for participants of the CPKD program compared to the control group (CPKD: 30% vs. ctrl: 10%; *p* < 0.001). Similarly, there were no significant differences in terms of students’ satisfaction with the thesis project, their supervision, daily work, or outcomes of the project. Nevertheless, students would conduct their MD thesis again (CPKD: 76.7% vs. ctrl: 63.3%; *p* = 0.163). Concerning the average number of publications, no differences were observed between both groups concerning first-author publications (CPKD: 0.62 vs. ctrl: 0.37; *p* = 0.245) and co-author publications (CPKD: 0.8 vs. ctrl: 0.45; *p* = 0.163). Congresses were significantly more often attended by CPKD students compared to students of the control group (CPKD: 68.3% vs. ctrl: 23.3%; *p* < 0.001). Moreover, students of the CPKD group gained on average significantly more rewards compared to students not participating in the program (CPKD: 38.3% vs. ctrl: 5%; *p* < 0.001). In contrast to the first matching, participants of the CPKD program did not reveal to conduct more often current scientific projects compared to students from the control group (CPKD: 50% vs. ctrl: 33.3%; *p* = 0.096). However, concerning their future scientific career as a clinician scientist, significantly more CPKD students pursued to participate in CSPs (CPKD: 45% vs. ctrl: 13.3%; *p* < 0.001). In line with the first matching, the number of students/physicians who already participate in a CSP is not differing between both groups. Similarly, also the average number of publications after the conduct of the MD thesis is not different between the CPKD and the control group (Table 6).

**Table 6:** Propensity score matched analysis based on age

	CPKD (*N* = 60)	control (*N* = 60)	*p*-value
Age, mean [years (IQT)]	27.00 (25, 29)	27.00 (25, 29)	0.998
Sex [*N* (%)]			
Male	32 (53.3)	19 (31.7)	0.027
Female	28 (46.7)	41 (68.3)	
Diverse	0 (0)	0 (0)	
Thesis [*N* (%)]			
Theoretical	2 (3.3)	9 (15.0)	0.058
Clinical	3 (5.0)	34 (56.7)	**< 0.001**
Experimental	58 (96.7)	18 (30.0)	**< 0.001**
Biomedical background of parents [*N* (%)]	28 (46.7)	30 (50.0)	0.855
Education background of parents [*N *(%)]	16 (27.1)	16 (28.1)	1.000
HS-GPA [mean (SD)]	1.33 (0.40)	1.41 (0.35)	0.258
SE-GPA [mean (SD)]	2.19 (0.56)	2.50 (0.84)	**0.02**
Number supervisors (> 2) [*N* (%)]	18 (30.0)	6 (10.0)	**< 0.001**
Satisfaction supervision [mean (SD)]	6.93 (3.14)	7.08 (3.25)	0.797
Satisfaction daily work [mean (SD)]	6.92 (2.77)	6.45 (2.82)	0.362
Satisfaction outcome [mean (SD)]	6.70 (3.03)	6.63 (3.01)	0.904
Conduct thesis again [mean (SD)]	46 (76.7)	38 (63.3)	0.163
Relevance MD thesis (> 5) [*N* (%)]	36 (60)	24 (40)	0.073
First-author publications [mean (SD)]	0.62 (1.51)	0.37 (0.69)	0.245
Co-author publications [mean (SD)]	0.80 (1.77)	0.45 (0.77)	0.163
Attendance congresses [*N* (%)]	41 (68.3)	14 (23.3)	**< 0.001**
Rewards [*N* (%)]	23 (38.3)	3 (5.0)	**<0.001**
Current projects [*N* (%)]	30 (50.0)	20 (33.3)	0.096
Participation CSP [*N* (%)]	7 (11.7)	2 (3.3)	0.166
Pursue CSP [*N* (%)]	27 (45.0)	8 (13.3)	**< 0.001**
Publications after thesis [mean (SD)]	51 (85.0)	47 (78.3)	0.479

The analyses of participant characteristics, subjective and objective outcome parameters, and the sustainable integration in scientific research are depicted. Significant *p*-values are highlighted in bold

Concerning the pursuit of CSPs the matched analysis showed that participants in CPKD group would five times more likely pursue a scientific carrier than participants in the control group (unadjusted odds ratio: 5.24, 95% CI [2.02, 15.03], *p* = 0.0002) (Table 7). This estimate did not take into account any other information. Adjusted estimates can be obtained from logistic regression. Logistic regression revealed that participants in CPKD group would 4.6 times more likely pursue a scientific carrier than participants in control group of the same age, HS-GPA, and SE-GPA (adjusted odds ratio: 4.605, 95% CI [1.78, 11.912], *p* = 0.002) (Table 8).

**Table 7:** Unadjusted odds ratio for the pursuit of a scientific career within a CSP

	Pursue CSP = Yes	Pursue CSP = No
CPKD (*n* = 60)	27	33
Control (*n* = 60)	8	52
Unadjusted odds ratio	5.24, 95% CI [ 2.02, 15.03], *p* = **0.0002 < 0.001**	

Significant *p*-values are highlighted in bold

**Table 8:** Adjusted odds ratio for the pursuit of a scientific career within a CSP

Risk factor	Estimate	95% CI	*p*-value
Group (Ref. = control)	4.605	[1.78, 11.912]	**0.002**
Age	0.89	[0.773, 1.024]	0.103
HS-GPA	0.333	[0.073, 1.522]	0.156
SE-GPA	0.535	[0.261, 1.093]	0.086

Significant *p*-values are highlighted in bold

## Discussion

During the course of their studies, more than half of German medical students conduct a doctoral thesis [10]. However, the scientific education received during the conduct of the doctoral thesis, supervision and quality of the work vary widely and greatly influence final results [28]. In order to improve the quality of the medical doctoral thesis, structured programs such as the CPKD have been developed. However, evidence concerning the impact of structured MD thesis programs on the conduct of the doctoral thesis is still lacking. Therefore, this study was performed to investigate the impact of a structured MD thesis program on the example of the MFD.

In this study, the impact of the CPKD was assessed by an online survey. The survey was sent to students who participated in the CPKD program as well as students who conducted their thesis independently of a structured program. In total, we contacted 3420 students or alumni of the MFD via email, of whom 370 filled in the questionnaire. The low response rate might be a result of switching institutions and inactivating email addresses. Furthermore, students might not be interested in the topic of their medical thesis anymore. In addition, the length and time to completion of our questionnaire might not be feasible for medical professionals. This might lead to an overrepresentation of current and in general more junior researchers in this study.

Our survey showed that the CPKD program had a positive impact on the sustainable integration of the students into the field of research based on current scientific projects and the pursuit of CSPs (Table 4). In contrast to other studies [29, 30], our study showed that participation in a structured thesis program inspires students to pursue a career as a clinician scientist by taking part in CSPs. While Pfeiffer et al. only investigated the motivation of participants of a structured thesis program [29], Claudia et al. also indirectly assessed the students’ career perspective by analyzing the participants’ pursuit of the “habilitation” [30]. CSPs do not only offer support in pursuing the next scientific career step, such as the “habilitation”, but also rather focus on continuous scientific growth and development of researchers [31].

In addition, our study revealed that a structured thesis program such as the CPKD affects the outcome of the doctoral thesis by increasing the attendance of congresses as well as the number of received research awards. A study conducted by Pfeiffer et al. showed that students participating in a doctoral thesis program have a higher intrinsic motivation compared to students who conduct their thesis independently from such a program [29]. In our study, this effect could have also influenced the results. However, it is also likely that participants in the CPKD were more often informed about possible congress attendance and application for research prizes. Due to the nature of self-application and the following selection process of the program, more intrinsically motivated students could have been included into the CPKD group. In line with this, the participants of the CPKD group were significantly younger and revealed a significantly better HS-GPA compared to the diverse and heterogeneous population of the control group.

CPKD students had a significantly higher number of supervisors compared to students not participating in such a program. This is due to the nature of the CPKD, assigning at least three supervisors per project within a thesis advisory committee (TAC) to allow different views and scientific rigor. Although the number of supervisors was significantly higher in the CPKD group, the mean number of supervisors differed from the prescribed number of three supervisors. This discrepancy could result from a misunderstanding of the question in the questionnaire because the term supervisor was not further specified. Therefore, we checked the protocols of all CPKD students who finished their thesis for their number of supervisors as they needed to record their TAC project meetings and name their TAC supervisors. Our investigations revealed that almost all CPKD students recorded three TAC supervisors indicating that the participants of the survey only recorded their daily supervisors and not the three TAC supervisors. Due to the impact of the CPKD program on the scientific output (Table 3) and the thesis results (Supplementary Fig. 1) changes in the guidelines for doctoral thesis occurred at the MFD. Recently, the number of supervisors changed for all students to be at least two. Interestingly, students’ satisfaction concerning their supervision, daily work during the thesis, and outcome were not significantly different between both groups. This could be due to a generally high acceptance of supervision by students at the MFD. Otherwise, it could imply that more than two supervisors do not automatically improve the students’ satisfaction concerning the supervision. However, a study conducted by Kuhnigk et al. showed that supervision is essential for the outcome of the thesis [32]. In general, students rated their satisfaction with supervision, daily work, and outcome quite favorable (Table 2). Importantly, the majority of students would also conduct their thesis again, attesting to the strength of scientific education and research of the MFD (Table 2).

Students of the CPKD program stated that participating in the program was beneficial for their scientific education. In line with this, other established graduate schools, for example in Frankfurt, have also shown to benefit student scientific education and were rated positively by students [33]. Students of similar programs at other universities did also significantly more often turn towards a research career [30]. Thus, our study supports the notion that early students’ participation in structured doctoral programs enhances the likelihood for pursuing clinician scientist programs later on. In line with this, students of the CPKD showed significantly higher continuation of scientific projects and currently perform research work. Additionally, participation in a structured thesis program was shown to favor a higher number of publications as well as in a higher impact factor of the published articles [34]. This was also indicated by our general analysis. Most strikingly, students of the CPKD achieved more rewards which may also attest to a higher scientific quality of their work.

Due to the selection process of the CPKD, genuine differences are observed between the CPKD and the control group in terms of age, HS-GPA, and SE-GPA. Therefore, we cannot exclude that the CPKD attracts a different type of students compared to the unstructured thesis leading to differences between both groups in the general analysis. The best way to reduce the bias that may have been induced by the selection of the participants of the CPKD was to conduct the propensity score matching. After matching, the SE-GPA was not differing anymore but the SE-GPA and the age were still different between both groups. Considering the propensity score matched analysis, most of the trends observed in the general analysis could be validated. However, some results such as the impact on the number of publications were not evident anymore. Due to differences concerning HS-GPA, SE-GPA, and age between the CPKD and the control group, even after matching based on the experimental type of thesis, we cannot rule out that the CPKD attracts a different type of students compared to the control group of unstructured MD thesis programs. To further reduce the potential bias induced by age differences, matching was conducted based on participant age. Even in the second matching, differences between both groups concerning SE-GPA were observed indicating that we could not completely remove the bias that was introduced by the participant selection for the CPKD. Therefore, a logistic regression analysis was conducted showing that participants of the CPKD would rather pursue a scientific career indicated by participating in a CSP. The adjusted odds ratio of 4.6 for the group comparison was statistically highly significant.

In contrast, this analysis also showed that neither participant age nor the results of HS-GPA and SE-GPA had a significant impact on the likelihood for participation in a clinician scientist program (Table 8). Although logistic regression was not performed for every item, we can conclude that programs such as the CPKD can beneficially support this selected intrinsically motivated subgroup of MD students and serve as an adjuvant support during the conduct of the thesis.

We can only assess the potential of a structured thesis program for the conduct of an experimental thesis based on our propensity score matched analysis. However, it is likely that such relationships could be also present in the context of statistical or clinical thesis projects. The investigation of these thesis types was out of the scope of this study. Consequently, additional research is needed, taking into account other types of doctoral thesis projects as they reveal individual challenges, in order to comprehensively evaluate the effects of structured thesis programs on the outcome of the doctoral thesis. In addition, structured thesis programs should be compared and evaluated on a nationwide basis with the aim of continuous improvement.

In our survey we did not request information from participants on their thesis grade. However, after we had completed the analysis of the survey and found significant differences of outcome parameters between the study groups (Tables 3 and 4), we became interested in the outcomes of the dissertation procedures as an additional outcome parameter. Data archived by the faculty with respect to the individual thesis grades awarded to medical dissertation students between 2011 and 2022 showed that the average thesis grade awarded differed significantly between participants of CPKD and control group (1.6 vs. 2.3; *p* < 0.0001, *n*_1_ = 64, *n*_2_ = 1679). Further, we analyzed the dataset with respect to individual thesis grades (Supplementary Fig. 1). These results comprehensively document a superior outcome with respect to thesis grades for the student group affiliated with the structured thesis program. We acknowledge that this does not reveal a causal relationship, but it points to a strong association, which would require further studies to better understand causal factors.

### Strength and Limitations

This study compared students in the structured thesis program of the CPKD to those conducting their thesis without such support at MFD using a non-validated survey. The study was a retrospective cohort and propensity score matched analysis. The retrospective aspect as well as the missing validation of the survey is limiting our ability to draw robust causal conclusions. Through the office of academic affairs, we contacted 3420 students, of whom 370 participated in our study. In 440 cases, the email addresses were invalid due to a switch of workplace. In addition, the groups differed significantly regarding age and this might have biased the responses. This difference probably occurred due to the large number of participants still conducting medical studies who have been in the CPKD compared to the control group with many participants who already concluded their studies. Furthermore, there were significant differences in the chosen type of medical thesis between both groups. The respective bias could be reduced, but not completely excluded, by introducing propensity score matched analyses. Due to the aim of the CPKD program, experimental, laboratory thesis projects were in the focus of the analysis.

However, the majority of students at MFD are conducting a clinical thesis rather than an experimental one. Therefore, our results may be biased toward the subgroup of experimental studies and it is unclear whether our findings may be generalized for clinical or theoretical thesis projects. Finally, we did evaluate one medical doctoral program in Germany. In future, it would be of interest to analyze results from different sites in a standardized manner.

## Conclusion

Our study revealed that structured MD thesis programs such as the CPKD significantly correlate with the students’ objective outcome of the MD thesis and help to inspire students to choose the track of academia. In general, based on our results, an MD thesis should be performed within structured programs. This leads to more achievements during the course of the thesis. More importantly, it supports medical students’ development in urgently needed clinician scientists to be able to perform high-level patient care and also excellent research. By elucidating the influence of structured thesis programs on the conduct of the doctoral thesis, this study provides valuable insights that can contribute to the further improvement of existing programs. Regarding the number of medical students per study year and the small fraction participating in the structured dissertation program at the MFD, we emphasize enlargement of the existing program and/or implementation of additional graduation programs. Ideally, such programs should address experimental, clinical, and statistical research likewise. Based on the results obtained in this study, we call for further efforts to strengthen research opportunities in German medical schools to develop internationally competitive medically qualified researchers who drive change and overcome the arising challenges in the field of medicine and beyond. The query posed by our title, “Do we need more structured MD thesis programs?” in our opinion, can be emphatically answered in the affirmative.

## Supplementary Information

Below is the link to the electronic supplementary material.

Supplementary file1 (DOCX 144 KB)

## Data Availability

Further data can be made available upon reasonable request to the corresponding author.
