# Supplementary material for: Do We Need More Structured MD Thesis Programs? A Propensity Score Matched Analysis of the Research Program at the Medical Faculty Dresden
Source: Med Sci Educ. 2024 Jun 13;34(5):1011–21. doi: 10.1007/s40670-024-02077-x (PMC11496407; doi:10.1007/s40670-024-02077-x)
Supplement: Supplementary file 1 — Supplementary file1 (DOCX 144 KB) [file 40670_2024_2077_MOESM1_ESM.docx]

**Supplementary Information (SI)**

**
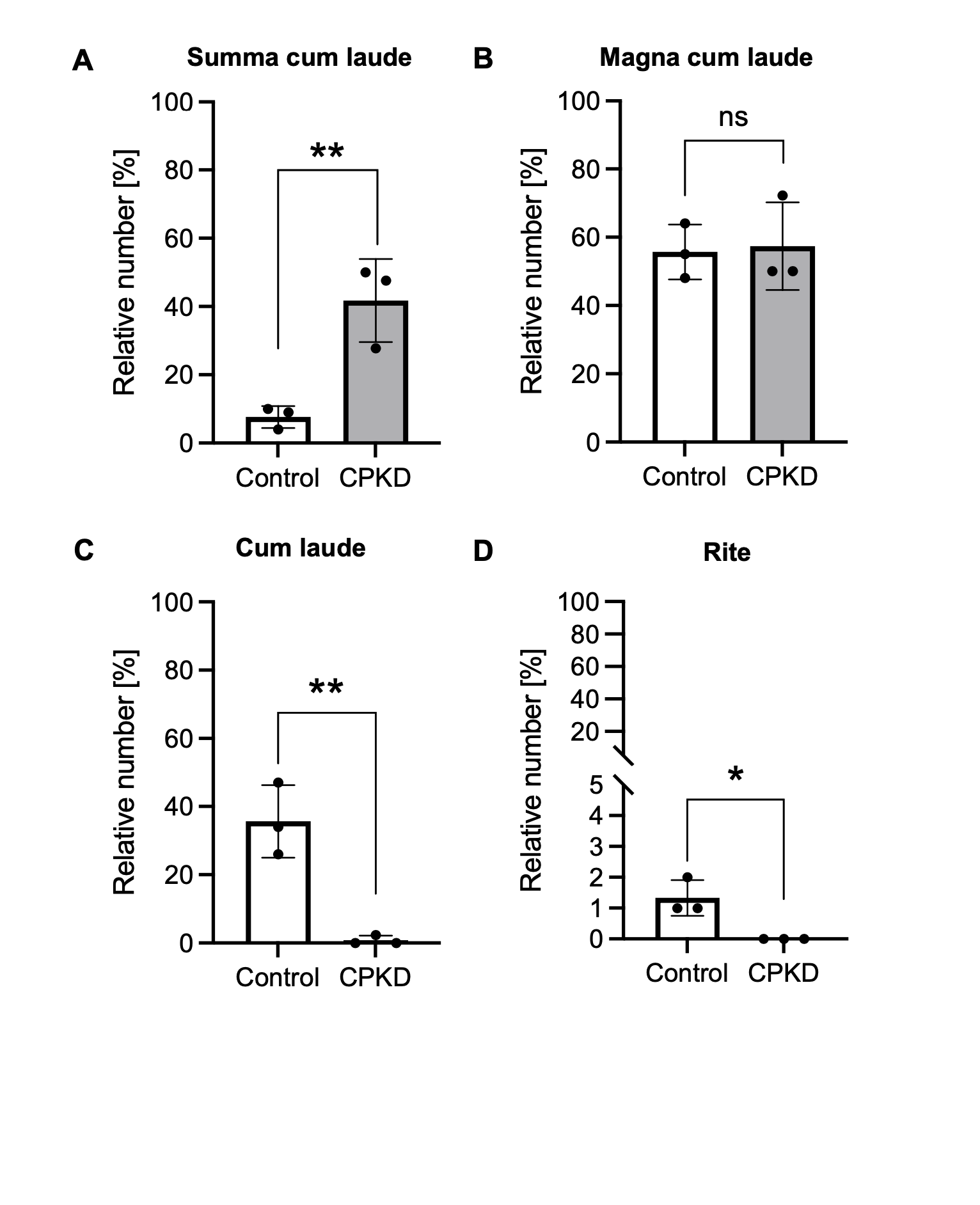
**

**Supplementary Fig. 1:** Analysis of the awarded individual thesis grades of CPKD students compared to the students of the control group. **(A)** Analysis of the relative number of the awarded thesis grade *summa cum laude* of the CPKD compared to the control group. **(B)** Analysis of the relative number of the awarded thesis grade *magna cum laude* of the CPKD compared to the control group. **(C)** Analysis of the relative number of the awarded thesis grade *cum laude* of the CPKD compared to the control group. **(D)** Analysis of the relative number of the awarded thesis grade *rite* of the CPKD compared to the control group. The mean value of the relative number of the individual thesis grade was calculated for the three different thesis types (experimental, clinical-conservative, clinical-operative). Statistical analysis was conducted using an unpaired Student's t-test. *P < 0.05; **P < 0.01.
